# A Novel Protocol for Model Calibration in Biological Wastewater Treatment

**DOI:** 10.1038/srep08493

**Published:** 2015-02-16

**Authors:** Ao Zhu, Jianhua Guo, Bing-Jie Ni, Shuying Wang, Qing Yang, Yongzhen Peng

**Affiliations:** 1 Key Laboratory of Beijing for Water Quality Science and Water Environmental Recovery Engineering, Engineering Research Center of Beijing, Beijing University of Technology, Beijing 100124, PR China; 2 Advanced Water Management Centre (AWMC), The University of Queensland, St Lucia, Brisbane, QLD 4072, Australia; 3 Tsinghua Holding Human Settlements Environment Institute, Beijing 100083, PR China

## Abstract

Activated sludge models (ASMs) have been widely used for process design, operation and optimization in wastewater treatment plants. However, it is still a challenge to achieve an efficient calibration for reliable application by using the conventional approaches. Hereby, we propose a novel calibration protocol, i.e. Numerical Optimal Approaching Procedure (NOAP), for the systematic calibration of ASMs. The NOAP consists of three key steps in an iterative scheme flow: i) global factors sensitivity analysis for factors fixing; ii) pseudo-global parameter correlation analysis for non-identifiable factors detection; and iii) formation of a parameter subset through an estimation by using genetic algorithm. The validity and applicability are confirmed using experimental data obtained from two independent wastewater treatment systems, including a sequencing batch reactor and a continuous stirred-tank reactor. The results indicate that the NOAP can effectively determine the optimal parameter subset and successfully perform model calibration and validation for these two different systems. The proposed NOAP is expected to use for automatic calibration of ASMs and be applied potentially to other ordinary differential equations models.

Activated sludge is the most widely used biological technology for treating domestic and industrial wastewater. After its development with 100 years of history, many novel and modified processes have been developed to meet the more and more stringent discharge and emission limits. However, most of operating systems are suffering some drawbacks, such as substantial energy consumption, excessive greenhouse gas emission, and labour-intensive industry. As a powerful tool, Activated Sludge Models (ASMs) have proven to be very useful in process design, operation and optimization^1^,^2^. To date, ASMs for the simulation of biological nutrients removal processes have been updated from the first version of ASM1 to more complicated extensions, including ASM2, ASM2d, and ASM3^3^, and further to the extended ASM3s^4^,^5^,^6^,^7^,^8^,^9^,^10^ in order to satisfy various requirements.

However, ASMs are large and overparameterized models in terms of having many stoichiometric and kinetic parameters. Some of the model parameters as well as the model structure have to be adjusted, since microbial community structure and dominant species can vary in different wastewater treatment systems with different influent characteristics or operation schemes^6^,^7^,^11^,^12^,^13^,^14^. In addition, the collected data from full-scale plants as well as pilot- or lab- scale reactors can hardly provide reliable estimations of all the parameters simultaneously due to the well-known problem of poorly identifiable parameters^15^,^16^,^17^,^18^,^19^,^20^. Thus, the approach to properly select the subsets of parameters for model calibration plays a crucial role on simulation results and model applications^21^,^22^,^23^,^24^,^25^.

Until now, substantial studies have been conducted to develop effective model calibration approaches, which can be distinguished into two major categories: the conventional experience-based approaches and the systems analysis approaches^22^. The experience-based approaches that were proposed by WERF, BIOMATH, STOWA, CALAGUA, HSG etc., import programmatic flow based on experts' knowledge and experience^22^,^26^,^27^. However, these calibration protocols require specific experimental designs and data processing methods to decouple the ASMs to be small and simple sub ones. The data for model calibration and validation may be from different feeding and operation conditions, and some of the parameters are fixed in order to obtain accurate estimation of the concerning ones. This approach might also ignore shifts in microbial community, eventually resulting in error estimations. Recently, the Good Modeling Practice (GMP) task group from IWA have emphasized that a standardized modeling procedure is needed to distinguish parameter subset that is identifiable with the available data for experience-based approaches^28^.

Systematical analysis approaches have attracted much more attention^15^,^18^,^29^,^30^,^31^,^32^, which mainly consist of parameter identification, sensitivity analysis, and error propagation^32^. Conventional parameter subset identifiability analyzing methods are mainly based on the local sensitivity analysis^21^,^22^. Recently, global sensitivity analysis (GSA) becomes a promising method for parameter identifiability analysis^33^,^34^,^35^, such as the Morris screening method^36^, Fourier Amplitude Sensitivity Testing (FAST), Extended-FAST, the Sobol's method^37^,^38^,^39^,^40^,^41^, and standardized regression coefficient (SRC) method^33^. Structural identification through Taylor series expansion^16^, generating series^42^, similarity transformation approach^43^ and differential algebra approach^17^,^44^, have been proposed in order to screen out the identifiable combinations. Based on the symbolic algebra system, structural identification has been applied to ASM-type simple sub-models successfully^16^,^17^. However, structural identification based on symbolic algebra system could be time consuming and unreliable due to the large number of parameters in ASMs. Therefore, it is highly desired to develop a numerical integrated approach by including both proper global parameter sensitivity analysis method and correlation analysis method for the identifiability of the parameters of the ASMs.

The main objective of this study is to develop a novel systematical approach, namely **N**umerical **O**ptimal **A**pproaching **P**rocedure (NOAP), for efficient calibration and validation of ASMs, taking an extended ASM3 as example. The NOAP integrates a new screening global parameter sensitivity analysis method (Derivative based Global Sensitivity Measures, DGSM) and a numerical correlation analysis method (based on the pseudo-global covariance matrix), with Genetic Algorithm (GA) as the global optimization (GO) method for parameter estimation. The validity and applicability of the approach for efficient model calibration are tested by independent experimental data from a Sequential Batch Reactor (SBR) activated sludge system^45^ and a Continuous Stirred-Tank Reactor (CSTR) activated sludge system^46^.

## Results

### Development of the NOAP approach

**Figure 1:**
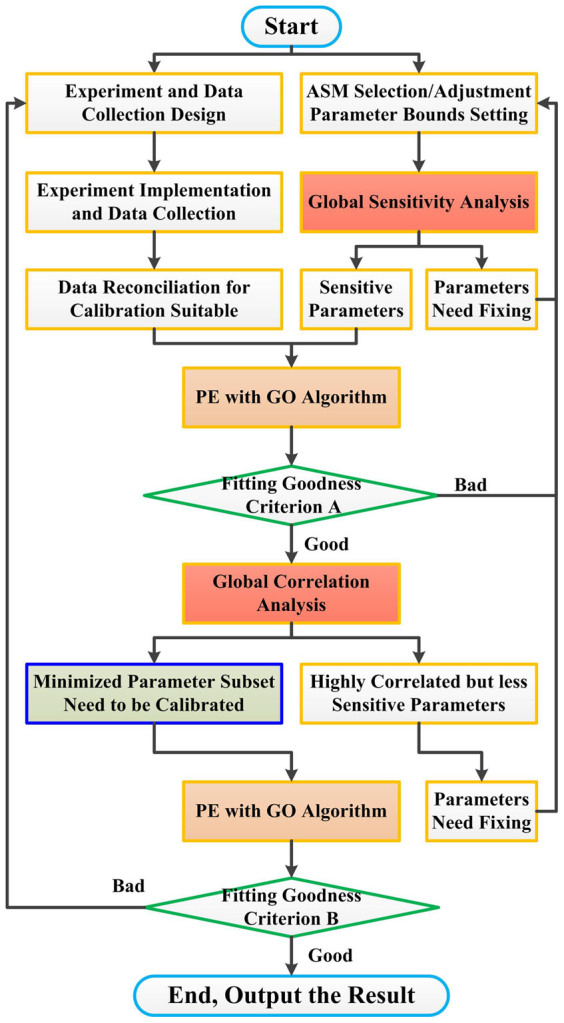
Scheme flow of the proposed NOAP (PE, parameter estimation; GO, global optimization).

The iterative algorithm of NOAP approach, as shown in the main scheme flow in Fig. 1, mainly consists of four steps in succession.

Step I: Model selection and data preparation. The user should firstly choose an appropriate model and extend it to satisfy the specific requirement from the current available models in previous studies^1^,^47^. Data collected from lab-, pilot-, and full-scale reactors are often used, but the amount and quality need reconciliation for the basic request of model calibration. The data collection and reconciliation can refer to the procedures suggested by Rieger et al.^28^.

Step II: GSA for factors fixing. Global parameter sensitivity analysis would be performed only if (i) the initial values of the state variables are set in a reasonable range based on data analysis and ii) parameter boundaries (lowest and highest) for global sensitivity analysis are proper. A fitting goodness criterion (such as A = 85%) between data and simulation results is used to judge parameter subset remained after factor fixing from DGSM analysis result. If the result is satisfied (R^2^ ≥ A), the parameters with lower sensitivity will be removed with trial, then repeating estimation trial until a proper small enough parameter subset is selected out. Otherwise, more non-sensitive ones should be added into the target subset one by one with the iterative parameter estimation trial. If parameters are all estimated simultaneously without a satisfied result, boundaries of the parameters or model structure might be improper and need further modifications.

Step III: For the scale reduced parameter subset, a pseudo-global correlation matrix is calculated. If the correlation coefficient of any parameter pair is high enough (>0.95 as example), then such parameter combinations are located to be highly correlated parameters due to the statistical average effect of the numerical algorithm. Afterwards, less interested members and correlation crossing ones (high correlated with not only just one parameter) can be fixed.

Step IV: The final parameter estimation is performed to check the efficiency of the procedure. Another fitting goodness criterion (such as B = 95%) is set to roughly test the quality of the observables' collected data.

### Preliminary model calibration based on conventional approach

According to Kaelin et al.^6^, the parameter subset [*μ_AOB_*, *μ_NOB_*, 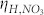, 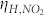, 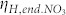, 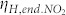] is firstly adopted and used to perform preliminary model calibration. Through this step, it is expected to: (1) determine whether the experimental data from the target system could be simulated by the proposed model; (2) suggest proper initial values of the state variables; and (3) verify directly whether the reported parameter subset from literature is efficient or not.

#### Scenario 1: SBR system

**Figure 2:**
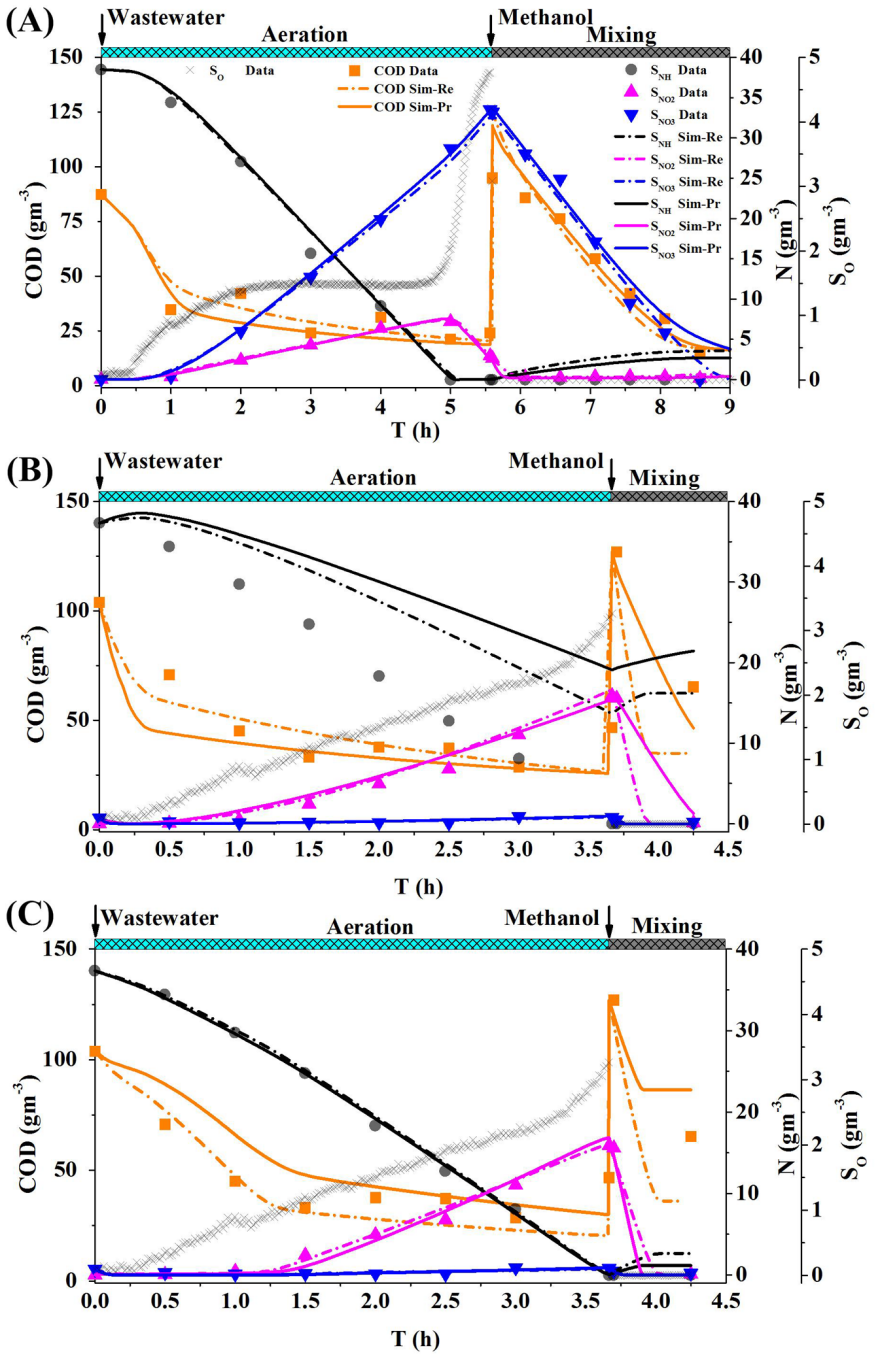
Calibration and validation results with the parameter subset suggested by the reference 6 and the proposed procedure NOAP for target SBR system. (A, Calibration results with the parameter subset recommended by the reference (Sim-Re)^6^ and the NOAP procedure (Sim-Pr); B, Validation for the calibration results as Fig. 2(a) presented, legends are the same as Fig. 2(a); C, Validation adjusted for the calibration with the parameter subset recommended by the reference^6^ and the proposed procedure NOAP with the suggestions of the global parameter sensitivity and correlation analysis results (Here 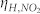 is added to the calibration subset), legends are the same as Fig. 2(a).)

As shown in Fig. 2(a), the simulated results (Sim-Re series) have a good fitness with the experimental data in terms of the profiles of COD, nitrite and nitrate concentrations. However, an unacceptable accumulation of ammonium during anoxic period is observed in the model simulations, which might be due to improper parameter values. Moreover, the validation (Sim-Re series) illustrated in Fig. 2(b) further verifies that the simulated ammonium results are much higher than the experimental data (R^2^(S_NH_ Sim-Re) = 0.5011).

Regarding the SBR system for nitrogen removal, the parameter subset selected from literature can partially calibrate the model to describe most of the experimental data. However, the extra efforts are needed to select other parameter subsets due to the failure of model validation.

#### Scenario 2: CSTR system

**Figure 3:**
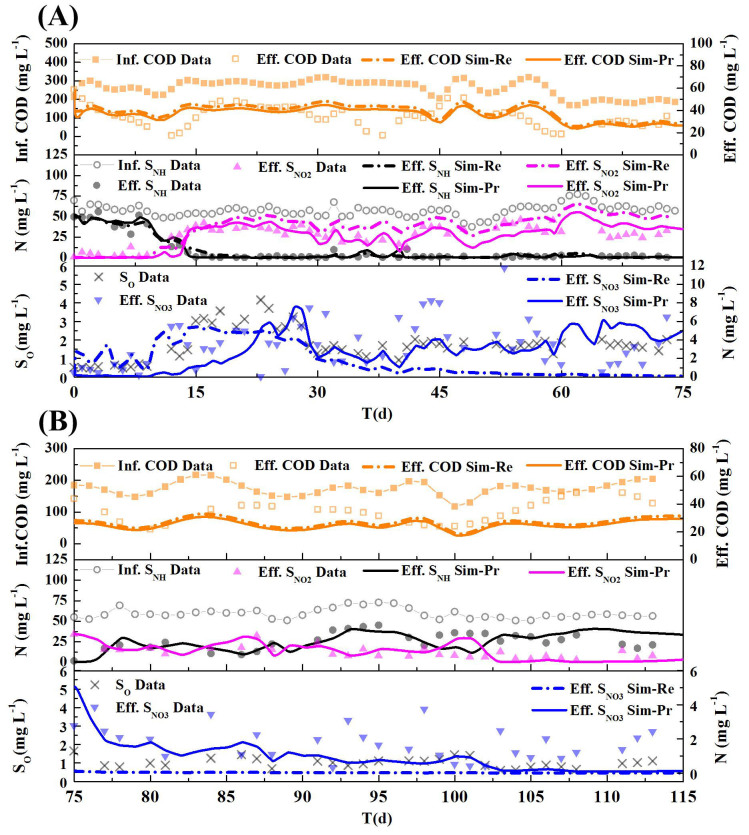
Calibration and validation with the parameter subset recommended by the reference^6^ and the proposed procedure NOAP for target CSTR system (A, Calibration results with the parameter subset suggested by the reference^6^ and the NOAP procedure (Sim-Pr); B, Validation for the calibration results as Fig. 3(a) presented.)

As shown in Fig. 3(a), the simulated (Sim-Re series) COD and ammonium concentrations in effluent fit well with the experimental data. However, the simulated nitrite concentrations are slightly higher than the experimental data (R^2^(Eff. S_NO2_ Sim-Re) = −0.3617), and the simulated nitrate concentrations are distinctly lower than the experimental data after the 30^th^ day (R^2^(Eff. S_NO3_ Sim-Re) = −1.0290). The validation results (Sim-Re series) in Fig. 3(b) suggest that all the measured nitrogen species concentrations (including ammonium, nitrite and nitrate) deviate distinctly from the experiment data (R^2^(Eff. S_NH_ Sim-Re) = −3.2151; R^2^(Eff. S_NO2_ Sim-Re) = −16.4224; R^2^(Eff. S_NO3_ Sim-Re) = −3.3828). This might be attributed to the improper parameter values of the dissolved oxygen (DO)-related kinetic coefficients, such as *K_H_*_, *O*2, *inh*_, *K_AOB_*_, *O*2_, and *K_NOB_*_, *O*2_. Hence, the model calibration is failed when applying the literature reported parameter subset^6^ into the CSTR system.

The preliminary calibration results from SBR and CSTR systems indicate that the parameter sensitivity vary across different activated sludge systems in the application of same model. Moreover, the parameter subset reported in literature could lead to poor model calibration results if fixing the remained parameters (e.g., the DO related parameters in Scenario 2). Thus, reliable parameter sensitivity analysis should be imported for model application in different activated sludge systems. It should be noted that the parameter subset reported in literature could be still useful for qualitatively model predictions, although it may not serve as a precise and quantitative predictor to match all experiment data.

### Parameter sensitivity and correlation analysis

According to the preliminary calibration and validation, it can be concluded that the extended ASM3 model could predict the data only if a proper parameter subset is selected. However, the parameter subset reported in literature^6^ cannot achieve satisfied calibration or validation results for both SBR and CSTR systems. Thus, numeric global parameter sensitivity and correlation analysis are performed to determine a more proper parameter subset.

**Figure 4:**
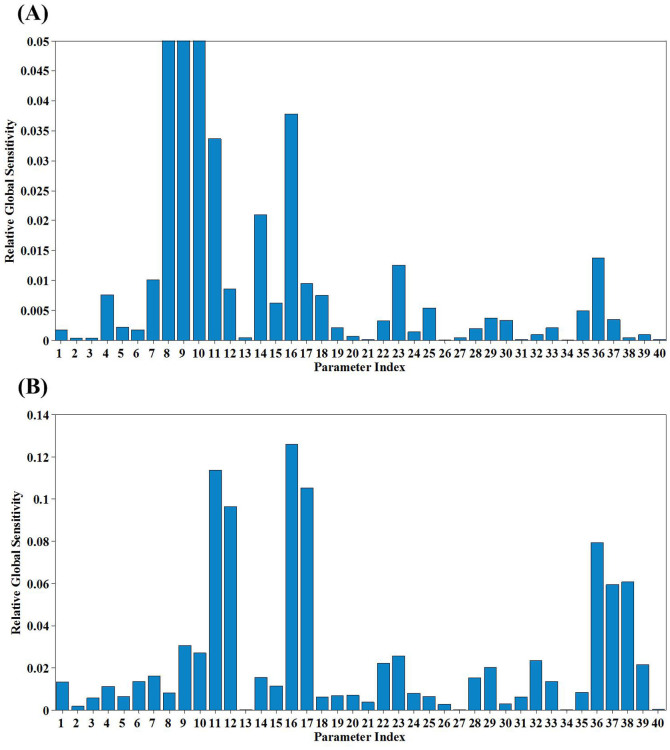
Global parameter sensitivity analysis results. (A, Parameter sensitivity analysis for the SBR system (parameter 8, 9 and 10 reached 0.20, 0.13 and 0.46, respectively; B, Parameter sensitivity analysis results for the CSTR system; The indexes and their corresponding parameters are listed as the follows: 1- *i_N_*_,*SS*_, 2- *i_N_*_,*XI*_, 3- *i_N_*_,*BM*_, 4- *f_XI_*, 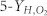, 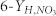, 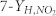, 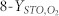, 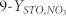, 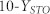, 11-*Y_AOB_*, 12-*Y_NOB_*, 13- *k_H_*, 14- *k_STO_*, 15-*μ_H_*, 16-*μ_AOB_*, 17-*μ_NOB_*, 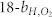, 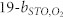, 20-*b_AOB_*, 21-*b_NOB_*, 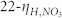, 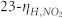, 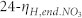, 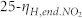, 26-*η_N_*_,*end*_, 27- *K_X_*, 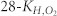, 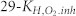, 30-*K_H,SS_*, 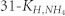, 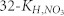, 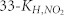, 34-*K_H,ALK_*, 35-*K_H,STO_*, 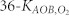, 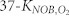, 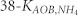, 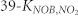, 40-*K_N,ALK_*. The meanings of each parameter can be found in Table S4, SI).

**Figure 5:**
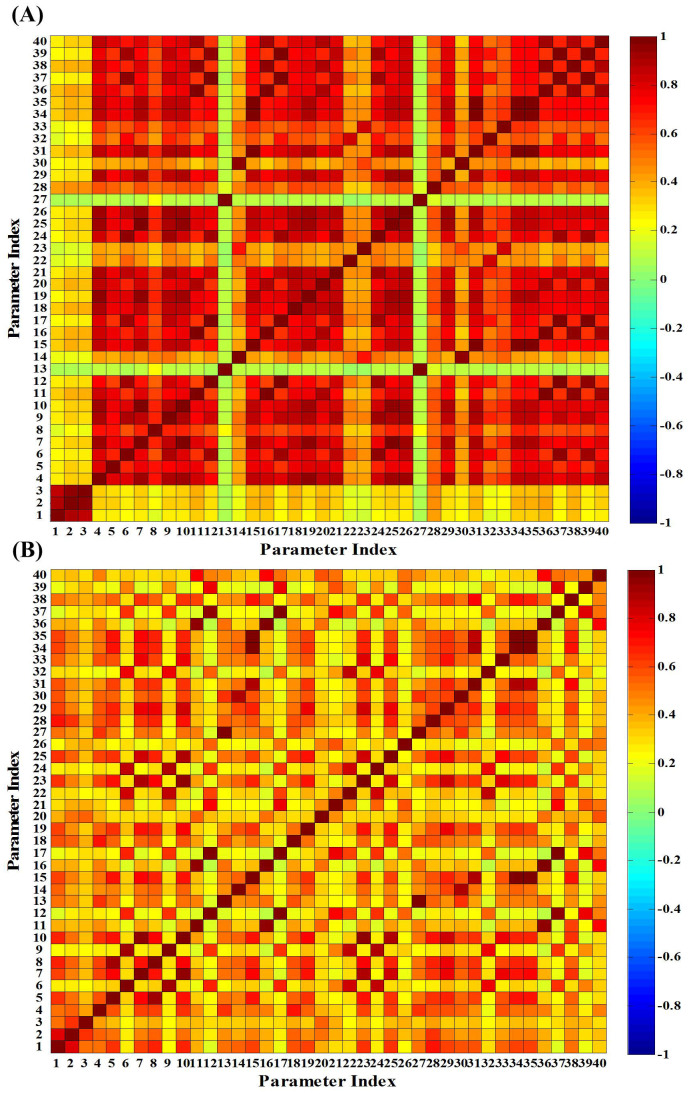
Global parameter correlation analysis results (color of the off-diagonal elements represents related parameters' correlation, between -1 and 1) (A, Parameter correlation analysis result matrix for the SBR system; B, Parameter correlation analysis result matrix for the CSTR system. The indexes and their corresponding parameters are the same with Fig. 4.)

In order to assess the identification of all the related parameters listed in Table S3 (SI), parameter sensitivity and correlation analysis are conducted for both SBR and CSTR systems. The proposed iterative trials (Fig. 1) are implemented in order to capture the best-fit parameter values. The results are shown in Fig. 4 and Fig. 5, respectively. It can be found that both sensitivity analysis and correlation analysis results are quite different between the SBR system and CSTR system. Furthermore, parameters indexed from 1 to 6 in Table S4 (SI) exhibit a similar low sensitivity for both SBR and CSTR systems, suggesting the composition coefficients could be fixed as default values due to their low sensitivity. However, most of the composition coefficients strongly depend on the influent wastewater characteristics. Therefore, it is recommended that the N content of biomass can be fixed as default values from references, while others (e.g. COD transformation fractions) need to be obtained experimentally.

For the SBR system, parameters including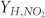, 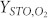, 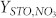, 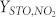, *Y_AOB_*, *k_STO_*, *μ_AOB_*, *μ_NOB_*, 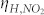
and 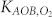
show high sensitivity, with their normalized relative influences reach 0.01 or even higher (Fig. 4(a)). Meanwhile, according to Fig. 5(a), the highly correlated parameter pairs are 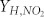 vs. 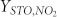 (R^2^ = 0.98), *Y_AOB_* vs. *μ_NOB_* (R^2^ = 0.99), *Y_AOB_* vs. 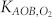 (R^2^ = 0.99), and *μ_AOB_* vs. 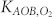 (R^2^ = 0.99). In addition, the sensitivity of 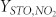 is much higher than 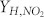 and *Y_AOB_*, and 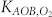 is highly correlated with other parameters, thus, 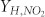, *Y_AOB_* and 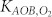 should be eliminated from the parameter subset. Finally, a parameter subset [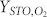, 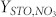, 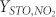, *k_STO_*, *μ_AOB_*, *μ_NOB_*, 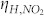] has been selected for the SBR system. Similarly, the parameter subset selected for CSTR system has been determined as [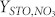, 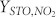, 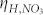, 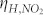, 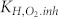, *K_H_*_,*NO*3_, 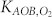, 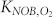]. The corresponding calibration and validation achieve satisfied results as shown in Fig. 2(b) and Fig. 3(b).

Optimal parameter subsets of SBR and CSTR systems for the calibration of the extended ASM3 are highly different. In the SBR system, three processes dominate the biological activities, which are storage of *S_S_* by heterotrophic organisms, growth of autotrophic organisms and denitrification via nitrite as the electron acceptor. These results are consistent with the experimental observations in the target SBR system, in which simultaneous nitrification and denitrification (SND) was observed and the nitrogen loss due to SND under aerobic condition was about 10 ~ 20 mg/L^45^. In contrast, for the CSTR system, the dominated biological activities include storage of *S_S_* by heterotrophic organisms, denitrification and inhibition of DO on heterotrophic denitrification and autotrophic growth. Hence, different optimal parameter subsets of SBR and CSTR systems may be mainly attributed to the different operation conditions, which lead to different dominant biological activities. From the modeling perspective, different initial values of the state variables, inputs like DO, influent COD and ammonium and the parameter boundaries would lead to different optimal parameter subsets.

### Parameter estimation and model validation based on the NOAP approach

In order to compare with the results obtained through the experience approach (i.e., preliminary calibration), the parameter subsets for SBR and CSTR systems determined by NOAP are further utilized for calibration and validation in this section. All the parameter estimation results can be found in Table 1.

#### Scenario 1: SBR system

As illustrated in Fig. 2(a), the subset of parameters determined by the NOAP can efficiently calibrate the extended ASM3 through the GO algorithm (see Sim-Pr series). The results indicate that a satisfied calibration could be obtained by different parameter subsets under the condition of limited data availability. Compared to the results of the traditional experience-based approach's calibration results (Fig. 2(a) Sim-Re series), the accumulation of ammonium during anoxic period in the NOAP calibration (Fig. 2(a) Sim-Pr series) is less, demonstrating a better fitting ability of the proposed NOAP approach. Furthermore, the nitrate reduction rate becomes slower when the biodegradable COD concentration is insufficient for denitrification. The simulated nitrate profile deviate slightly from the trend of the experimental nitrate concentrations, which indicates the parameter subset determined by the NOAP is more sensitive than that suggested by the traditional experience-based approach.

Although the calibration is successfully achieved, the initial validation (Fig. 2(b) Sim-Pr series) is still failed (R^2^(S_NH_ Sim-Pr) = 0.0997), similar with the validation by the experience-based parameter subset (Fig. 2(b) Sim-Re series). However, the proposed NOAP provides possible solutions to improve the simulation performance. After the iterative parameter subset selection procedure by NOAP, the parameter 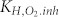 is further added into the parameter subset for re-calibration of the model. As shown in Fig. 2 (c), the performance of re-calibration for both parameter subsets (suggested by the traditional experience-based approach and the NOAP, respectively) are improved after adding the parameter 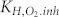 in the parameter subset. In addition, the parameter subset [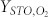, 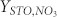, 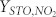, *k_STO_*, *μ_AOB_*, *μ_NOB_*, 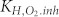, 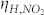] determined by the NOAP can reach a better fitting goodness, as the simulated ammonium concentrations match better with the original data during the anoxic period (Fig. 2 (c)). Thus, the proposed NOAP demonstrates its ability to provide additional information for improving model calibration.

#### Scenario 2: CSTR system

As present as Fig. 3, the calibration results (see Sim-Pr series) with parameter subset determined by the NOAP procedure achieve a much better fitting goodness with the experimental data (as shown in Fig. 3(a)). The validation of the calibrated parameters (Fig. 3(b)) also shows a good fitting except the period from day 99 to day 101, in which a lower ammonium and higher nitrite concentrations are predicted. The reason for such phenomena might result from the use of DO as an input for simulation and the DO concentrations during the period are higher than the real concentrations. It can be concluded that parameter subset [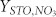, 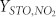, 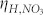, 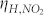, 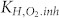, *K_H_*_,*NO*3_, 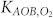, 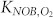] selected by the proposed NOAP could achieve a better fitting, with the same GO algorithm.

## Discussion

In this work, a novel NOAP approach for the efficient calibration of activated sludge models with limited available data has been proposed. The proposed NOAP integrates a new numerical global parameter sensitivity analysis method (DGSM) for factor fixing and a numerical pseudo-global parameter correlation analysis method for non-identifiable parameter detection to determine the optimal parameter subset for model calibration. The validity and applicability of the approach for efficient model calibration is confirmed by two different activated sludge systems (SBR and CSTR systems). The model calibration results suggested that the optimal sensitive parameter subsets of the SBR and CSTR system are different despite with the same extended ASM3 model to calibrate. Even with the same biomass collected from a municipal WWTP, two SBR reactors finally result in different optimal parameter subsets due to different operational conditions. The results indicate that the parameter subsets determined by NOAP can tail with the state variation of the system. This outstands from the experience-based procedures in calibrating dynamic systems as activated sludge systems whose parameters and structure can vary gradually, which would facilitate modeling automation a lot to support more optimization applications of WWTP.

The optimal parameter subset is different and specific for various systems, because of differences in environmental conditions, influent characteristics, operation modes and biomass population. As a black-box method, conventional experience-based model calibration procedures construct a mapping relation between data and model parameter subset, in which mapping routines are based on experts' empirical knowledge. However, uncertainty would be inevitable due to the arbitrary subset selection. For example, these risks may be distinctly enlarged when modeling the dynamic SBR scenario. An efficient calibration procedure is not only simple to fit the trend of historical data by manually selecting a parameter subset, but should be competent for optimal parameter subset determination, with the aids of efficient parameter estimation algorithms. The proposed NOAP in this study could be a promising alternative to fulfill the described demands. Since the global sensitivity analysis possesses the ability to evaluate uncertainty impact of the concerning factors on the model outputs scientifically. In fact, through factors fixing, parameter estimation is performed for top uncertainty introducers, which would reduce outputs' uncertainty in maximization. Consequently, through parameter estimation of subset determined by the global sensitivity analysis, the possibility of successful model calibration and prediction can be maximized. Furthermore, parameter subset optimized by numerical global parameter correlation analysis would enhance the success of calibration and validation. Simultaneously, the proposed NOAP method can quickly capture the shifts of system states through continuous updating of the known Factors' values and unknown Factors' boundary variations. Thus, the proposed NOAP is a promising and useful tool for the efficient calibration of ASMs and could potentially apply to other ordinary differential equations models.

Currently, the proposed NOAP procedure is a decision-helping tool, rather than an automatic protocol. In fact, it is expected to develop a fully automatic calibration procedure in future. Firstly, the automatic calibration procedure should recognize the optimal parameter subset for any models, and organize efficient parameter estimation automatically and robustly, through efficient numerical or symbolic algebra calculation approaches. Secondly, automatic methods are available for optimal experiments design and data collection, when uncertainty analysis of the parameter estimated is necessary in case of improving confidence intervals. Moreover, this calibration procedure can provide implementation procedure for automatic modification, selection and extension of model structure, when both model calibration and validation failed after data optimization. Most importantly, the calibrated model cannot only monitor and predict the overall process dynamics, but also facilitate the operators to achieve optimal control of a target system.

## Methods

### Parameter estimation, parameter sensitivity and correlation analysis

The parameter estimation is a critical step of the model calibration process. Stochastic global optimization algorithms can find the global minimum of the objective function given by equation (1)^48^,^49^, where ***y_0_*** = [ *COD*(0) *S_NH_*_4_(0) *S_NO_*_2_(0) *S_NO_*_3_(0) ]*^T^* as the example. Fitting goodness criterion function for each observable is given by equation (2). Characterizations and explanations of the symbols presented in this section can be found in the Table S1 (Supporting Information, SI).
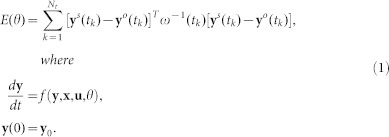

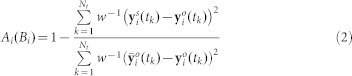
GA possesses the advantages of easy implementation and mature codes to reuse compared to other resembled technologies^50^,^51^,^52^. In this study, the MATLAB R2010a (Global Optimization Toolbox) is referred as the numerical function implementation of GA (The Mathworks Inc. USA).

To realize factor fixing, a Derivative based Global Sensitivity Measures (DGSM) method is introduced to perform the global sensitivity analysis^53^,^54^. By comparisons among DGSM, Morris and Sobol's method, it indicates that: a) DGSM shows much higher convergence rate and more accurate than Morris method for non-monotonic functions; b) there is a link between DGSM and Sobol' global sensitivity indices, but the computational time required for numerical evaluation of DGSM measures is many orders of magnitude lower^55^,^56^. Essentially, the DGSM method is based on the local sensitivity measure, but perform an average of the local sensitivity measure throughout the parameter space by introducing Quasi Monte Carlo sampling methods. The relative time varying sensitivity matrix is described as the following equation (3).
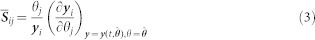
Average 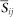 over the parameter space using Quasi Monte Carlo sampling methods, a measure can be defined as the equation (4).
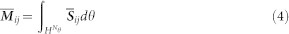
The numerical computation format can be expressed as the equation (5).
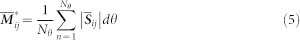
To overcome the time varying character, the global sensitivity analysis indices of each parameter are defined as equation (6).
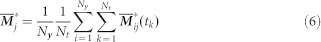
About the global Correlation Analysis, a pseudo-global correlation matrix is introduced^54^. The local Fisher Information Matrix (FIM) is described as equation (7).
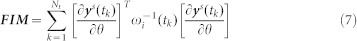
The derivative covariance matrix is an approximation of the inverse of the FIM as equation (8).
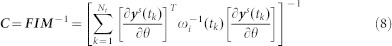
To introduce the pseudo-global covariance matrix, the local covariance matrix needs to be averaged throughout the parameter space like DGSM done with each objective function's value as the weight as equation (9).
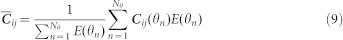
According to the pseudo-global covariance matrix, the correlation matrix is defined as equation (10).
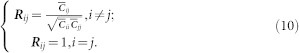
Based on the equations (6) and (10), parameter sensitivity ranking order and correlation relationships would be produced systematically.

### Activated sludge model and experimental data for NOAP testing

An extended ASM3 for two-step nitrification and denitrification^6^ is used for verifying the proposed procedure. The model inherits the basic mechanism settings of ASM3, in the frame of “Hydrolysis – Storage – Growth - Respiration”, nitrification and denitrification are extended to meet current need of description for main intermediate product – nitrite. The kinetic equations and stoichiometric matrix are presented in Table S2 and Table S3 (SI), respectively. Model structure and parameter settings are kept as the original for possibility of results comparison.

In addition, basic stoichiometric and kinetic parameters related information is presented in the Table S4 (SI), as well as parameter boundaries for GSA and parameter estimation. The validity and applicability of the approach is confirmed using experimental data obtained with two independent wastewater treatment systems, including SBR^45^ and CSTR^46^, respectively.

Experimental data of the SBR related scenario were collected from two reactors with a working volume of 14 L. Both reactors were seeded with the same inoculum from a full-scale municipal WWTP, but operated in different modes^45^. One was operated with the complete nitrification mode, while the other one was operated with the partial nitrification mode. The complete nitrification mode was operated in the aerobic-anoxic scheme with extensive aeration. Each cycle of the aerobic-anoxic scheme consisted of 3 min feeding, aeration, anoxic phase, 1 h settling, 6 min decanting, and 1 min idling. Aeration was still provided for another 0.5 h after the ammonium has been completely oxidized to nitrite, which would offer ideal environment for the nitrite-oxidizing bacteria to oxidize the nitrite successively to nitrate completely. The data from the complete nitrification reactor are used for the preliminary model calibration because the kinetic properties of the microorganisms in the system can be properly captured by these data series. In addition, the reactor with partial nitrification mode was also operated in the aerobic-anoxic scheme, but aeration duration was controlled through a real-time control system. The data from the partial nitrification reactor with obvious nitrite accumulation are applied to validate the preliminary model calibration results^45^.

The lab-scale CSTR was set up to achieve partial nitritation. The reactor had an effective reaction volume of 4 L, followed by an clarifier with a working volume of 3.5 L. Sludge retention time (SRT) was kept at 12 days by wasting sludge from the secondary clarifier. Experimental data of the CSTR related scenario illustrated an obvious effect of DO on the nitrite accumulation in the CSTR system, which could be used to identify and estimate DO related switching function parameters. DO play an important role in biological nitrogen removal processes. Controlling DO at a proper level can not only reduce energy consumption, but also favor the partial nitrification for nitrogen removal via nitrite^57^. During the simulation in this case, experimental data from 113-day operation of the CSTR are divided into two groups. One group (0^th^ to 75^th^ day) is used for the preliminary calibration, while the other group (75^th^ to 113^th^ day) is used for the validation.

## Author Contributions

J.G. and Y.P. conceived and designed the experiments; A.Z. and J.G. performed the modelling and analyzed the data; S.W., B.-J.N., Q.Y. contributed materials/analysis tools; A.Z., J.G. and B.-J.N. wrote the paper. All authors reviewed the manuscript.

## Supplementary Material

Supplementary Information Supporting information

## Figures and Tables

**Table 1:** Parameter estimation results of each model calibrations after global optimization using Genetic Algorithm

Subset suggested by reference			Subset selected by NOAP					
	Value		First calibration of SBR scenario		Re-calibration of SBR for improvement		CSTR scenario	
Parameter	SBR scenario	CSTR scenario	Parameter	Value	Parameter	Value	Parameter	CSTR scenario
*μ_AOB_*	0.78 d^−1^	0.38 d^−1^	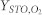	0.53 g COD/g COD	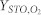	0.53 g COD/g COD	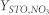	0.50 g COD/g COD
*μ_NOB_*	0.73 d^−1^	0.34 d^−1^	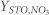	0.44 g COD/g COD	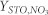	0.44 g COD/g COD	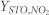	0.36 g COD/g COD
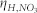	0.11	0.08	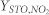	0.24 g COD/g COD	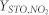	0.24 g COD/g COD	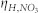	0.42
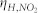	0.99	0.94	*k_STO_*	4.37 d^−1^	*k_STO_*	4.37 d^−1^	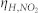	0.52
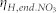	0.86	0.05	*μ_AOB_*	1.02 d^−1^	*μ_AOB_*	1.02 d^−1^	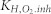	0.73 g O_2_ m^−3^
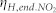	0.95	0.98	*μ_NOB_*	0.76 d^−1^	*μ_NOB_*	0.76 d^−1^	*_KH,NO_*_3_	5.69 g N m^−3^
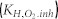	(18.44 g O_2_ m^−3^)	^−^	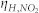	0.78	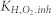	2.85 g O_2_ m^−3^	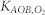	3.11 g O_2_ m^−3^
-	-	-	-	-	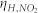	0.78	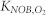	2.47 g O_2_ m^−3^
