# Supplementary material for: A Novel Protocol for Model Calibration in Biological Wastewater Treatment
Source: Sci Rep. 2015 Feb 16;5:8493. doi: 10.1038/srep08493 (PMC4329560; doi:10.1038/srep08493)
Supplement: Supplementary Information — Supporting information [file srep08493-s1.pdf]

Supporting Information

Title:

A Novel Protocol for Model Calibration in Biological Wastewater Treatment

Authors:

Ao Zhu[a,c], Jianhua Guo[a,b,*], Bing-Jie Ni[b], Shuying Wang[a], Qing Yang[a], Yongzhen Peng[a,*]

[a]*Key Laboratory of Beijing for Water Quality Science and Water Environmental Recovery Engineering, Engineering Research Center of Beijing, Beijing University of Technology, Beijing* 100124, *PR China*

[b]Advanced Water Management Centre (AWMC), The University of Queensland, St Lucia, Brisbane, QLD 4072, Australia

[c]Tsinghua Holding Human Settlements Environment Institute, *Beijing* 100083, *PR China*

*Corresponding authors:

Jianhua Guo, Phone: +86-10-67392627; fax: +86-10-67392627; e-mail: gjh@bjut.edu.cn.

Yongzhen Peng, Phone: +86-10-67392627; fax: +86-10-67392627; e-mail: pyz@bjut.edu.cn

Contents:

Table S1. Characterizations explanations of the symbols.

Table S2. Summary of the process rates equations for related processes.

Table S3. Stoichiometric matrix for the extended processes and selected compounds.

Table S4. Kinetic and stoichiometric parameters at 20 ℃.

Table S1. Characterizations explanations of the symbols.

Symbol	Characterization
$\boldsymbol{x}$	State variables of the Ordinary Differential Equations(ODEs)
$\boldsymbol{u}$	Input variables of the ODEs
$\boldsymbol{y}$	Outputs of the ODEs , such as$[COD\ S_{NH4} S_{NO2}\ S_{NO3}]^T$, $\boldsymbol{y}^s$ the simulation ones and $\boldsymbol{y}^o$ the observed measurements
$\boldsymbol{\theta}$	Parameter set of the ODEs
$\boldsymbol{\omega}$	the square noise variance matrix, whose inverse form $\boldsymbol{\omega}^{-1}$ acts as the weights
$E(\boldsymbol{\theta})$	Objective function of the parameter estimation
$\boldsymbol{y}_i$, N_y	the *i*-th state variable or output, the number of outputs
$\boldsymbol{\theta}_j$, $N_{\boldsymbol{\theta}}$	the *j*-th parameter in the parameter set, the number of parameters
t_k, N_t	the *k*-th measuring time, the number of measuring times
$\overline{\boldsymbol{S}}$	the local relative time varying sensitivity matrix at parameter point $\hat{\theta}$
$\overline{\boldsymbol{M}}$	the global relative time varying sensitivity matrix over the bounds-defined parameter space
$\boldsymbol{FIM}$	the local Fisher Information Matrix
$\boldsymbol{C}$	the local covariance matrix derived from the $\boldsymbol{FIM}$
$\overline{\boldsymbol{C}}$	the pseudo-global covariance matrix, derived from $\boldsymbol{C}$ and $E(\boldsymbol{\theta})$
$\boldsymbol{R}$	the pseudo-global correlation matrix, derived from $\overline{\boldsymbol{C}}$

Table S2. Summary of the process rates equations for related processes.

		j	Process	Modified process rate equation
Heterotrophic organisms	1	p1	Hydrolysis	$k_H \frac{X_S / X_H}{K_X + X_S / X_H} X_H$
	2	p2	Aerobic Storage of S_S	$k_{STO} \frac{S_O}{K_{H,O_2} + S_O} \frac{S_S}{K_{H,SS} + S_S} X_H$
	3	p3(a)	Anoxic Storage of S_S NO_3-NO_2	$k_{STO} \eta_{H,NO_3} \frac{K_{H,O_2inh}}{K_{H,O_2inh} + S_O} \frac{S_S}{K_{H,SS} + S_S} \frac{S_{NO_3}}{K_{H,NO_3} + S_{NO_3}} X_H$
	4	p3(b)	Anoxic Storage of S_S NO_2-N_2	$k_{STO} \eta_{H,NO_2} \frac{K_{H,O_2inh}}{K_{H,O_2inh} + S_O} \frac{S_S}{K_{H,SS} + S_S} \frac{S_{NO_2}}{K_{H,NO_2} + S_{NO_2}} X_H$
	5	p4	Aerobic Growth of X_H	$\mu_H \frac{S_O}{K_{H,O_2} + S_O} \frac{S_{NH_4}}{K_{H,NH_4} + S_{NH_4}} \frac{S_{ALK}}{K_{H,ALK} + S_{ALK}} \frac{X_{STO} / X_H}{K_{H,STO} + X_{STO} / X_H} X_H$
	6	p5(a)	Anoxic Growth of X_H NO_3-NO_2	$\mu_H \eta_{H,NO_3} \frac{K_{H,O_2inh}}{K_{H,O_2inh} + S_O} \frac{S_{NH_4}}{K_{H,NH_4} + S_{NH_4}} \frac{S_{ALK}}{K_{H,ALK} + S_{ALK}} \frac{X_{STO} / X_H}{K_{H,STO} + X_{STO} / X_H} \frac{S_{NO_3}}{K_{H,O_2inh} + S_{NO_3}} X_H$
	7	p5(b)	Anoxic Growth of X_H NO_2-N_2	$\mu_H \eta_{H,NO_3} \frac{K_{H,O_2inh}}{K_{H,O_2inh} + S_O} \frac{S_{NH_4}}{K_{H,NH_4} + S_{NH_4}} \frac{S_{ALK}}{K_{H,ALK} + S_{ALK}} \frac{X_{STO} / X_H}{K_{H,STO} + X_{STO} / X_H} \frac{S_{NO_2}}{K_{H,O_2inh} + S_{NO_2}} X_H$
	8	p6	Aerobic Endog. Resp. of X_H	$b_{H,O_2} \frac{S_O}{K_{H,O_2} + S_O} X_H$
	9	p7(a)	Anoxic Endog. Resp. of X_H NO_3-NO_2	$b_{H,O_2} \eta_{H,endNO_3} \frac{K_{H,O_2inh}}{K_{H,O_2inh} + S_O} \frac{S_{NO_3}}{K_{H,NO_3} + S_{NO_3}} X_H$
	10	p7(b)	Anoxic Endog. Resp. of X_H NO_2-N_2	$b_{H,O_2} \eta_{H,endNO_2} \frac{K_{H,O_2inh}}{K_{H,O_2inh} + S_O} \frac{S_{NO_2}}{K_{H,NO_2} + S_{NO_2}} X_H$
	11	p8	Aerobic Endog. Resp. of X_{STO}	$b_{STO,O_2} \frac{S_O}{K_{H,O_2} + S_O} X_{STO}$
	12	p9(a)	Anoxic Endog. Resp. of X_{STO} NO_3-NO_2	$b_{STO,O_2} \eta_{H,endNO_3} \frac{K_{H,O_2inh}}{K_{H,O_2inh} + S_O} \frac{S_{NO_3}}{K_{H,NO_3} + S_{NO_3}} X_{STO}$
	13	p9(b)	Anoxic Endog. Resp. of X_{STO} NO_2-N_2	$b_{STO,O_2} \eta_{H,endNO_2} \frac{K_{H,O_2inh}}{K_{H,O_2inh} + S_O} \frac{S_{NO_2}}{K_{H,NO_2} + S_{NO_2}} X_{STO}$
AOB	14	p10(a)	Aerobic Growth of X_{AOB}, Nitritation	$\mu_{AOB} \frac{S_O}{K_{AOB,O_2} + S_O} \frac{S_{NH_4}}{K_{AOB,NH_4} + S_{NH_4}} \frac{S_{ALK}}{K_{N,ALK} + S_{ALK}} X_{AOB}$
	15	p11(a)	Aerobic End. Resp. of X_{AOB}	$b_{AOB} \frac{S_O}{K_{H,O_2} + S_O} X_{AOB}$
	16	p12(a)	Anoxic End. Resp. of X_{AOB}	$b_{AOB} \eta_{N,end} \frac{K_{H,O_2inh}}{K_{H,O_2inh} + S_O} \frac{S_{NO_3}}{K_{H,NO_3} + S_{NO_3}} X_{AOB}$
NOB	17	p10(b)	Aerobic Growth of X_{NOB}, Nitratation	$\mu_{NOB} \frac{S_O}{K_{NOB,O_2} + S_O} \frac{S_{NH_4}}{K_{H,NH_4} + S_{NH_4}} \frac{S_{NO_2}}{K_{NOB,NO_2} + S_{NO_2}} \frac{S_{ALK}}{K_{N,ALK} + S_{ALK}} X_{NOB}$
	18	p11(b)	Aerobic End. Resp. of X_{NOB}	$b_{NOB} \frac{S_O}{K_{H,O_2} + S_O} X_{NOB}$
	19	p12(b)	Anoxic End. Resp. of X_{NOB}	$b_{NOB} \eta_{N,end} \frac{K_{H,O_2inh}}{K_{H,O_2inh} + S_O} \frac{S_{NO_3}}{K_{H,NO_3} + S_{NO_3}} X_{NOB}$
	20		Aeration	$e^{0.024(T-20)} \cdot K_L a\_20 \cdot (S_{O,SAT} - S_O)$

Table S3. Stoichiometric matrix for the extended processes and selected compounds.

	j	Process	S_O	S_S	S_{NH}	S_{NO2}	S_{NO3}	S_{N2}	S_{ALK}	S_I	X_I	X_H	X_S	X_{STO}	X_{AOB}	X_{NOB}
1	p1	Hydrolysis	0	1-f_{SI}	0	0	0	0	$\frac{i_{N,XS}-f_{SI}\cdot i_{N,SI}-(1-f_{SI})\cdot i_{N,SS}}{14}$	f_{SI}	0	0	-1	0	0	0
2	p2	Aerobic Storage of S_S	$Y_{STO,O_2}-1$	-1	$i_{N,SS}$	0	0	0	$\frac{i_{N,SS}}{14}$	0	0	0	0	Y_{STO,O_2}	0	0
3	p3(a)	Anoxic Storage of S_S NO_3-NO_2	0	-1	$i_{N,SS}$	$\frac{1-Y_{STO,NO_3}}{1.14}$	$-\frac{1-Y_{STO,NO_3}}{1.14}$	0	$\frac{i_{N,SS}}{14}$	0	0	0	0	Y_{STO,NO_3}	0	0
4	p3(b)	Anoxic Storage of S_S NO_2-N_2	0	-1	$i_{N,SS}$	$-\frac{1-Y_{STO,NO_2}}{1.72}$	0	$\frac{1-Y_{STO,NO_2}}{1.72}$	$\frac{i_{N,SS}-(Y_{STO,NO_2}-1)/1.72}{14}$	0	0	0	0	Y_{STO,NO_2}	0	0
5	p4	Aerobic Growth of X_H	$1-1/Y_{H,O_2}$	0	$-i_{N,BM}$	0	0	0	$-\frac{i_{N,BM}}{14}$	0	0	1	0	$-1/Y_{H,O_2}$	0	0
6	p5(a)	Anoxic Growth of X_H NO_3-NO_2	0	0	$-i_{N,BM}$	$\frac{1-Y_{H,NO_3}}{1.14\cdot Y_{H,NO_3}}$	$-\frac{1-Y_{H,NO_3}}{1.14\cdot Y_{H,NO_3}}$	0	$-\frac{i_{N,BM}}{14}$	0	0	1	0	$-1/Y_{H,NO_3}$	0	0
7	p5(b)	Anoxic Growth of X_H NO_2-N_2	0	0	$-i_{N,BM}$	$-\frac{1-Y_{H,NO_2}}{1.72\cdot Y_{H,NO_2}}$	0	$\frac{1-Y_{H,NO_2}}{1.72\cdot Y_{H,NO_2}}$	$\frac{(Y_{H,NO_2}-1)/(1.72\cdot Y_{H,NO_2})-i_{N,BM}}{14}$	0	0	1	0	$-1/Y_{H,NO_2}$	0	0
8	p6	Aerobic Endog. Resp. of X_H	-(1- f_{XI})	0	$-f_{XI}\cdot i_{N,XI}+i_{N,BM}$	0	0	0	$\frac{-f_{XI}\cdot i_{N,XI}+i_{N,BM}}{14}$	0	f_{XI}	-1	0	0	0	0
9	p7(a)	Anoxic Endog. Resp. of X_H NO_3-NO_2	0	0	$-f_{XI}\cdot i_{N,XI}+i_{N,BM}$	$\frac{1-f_{XI}}{1.14}$	$-\frac{1-f_{XI}}{1.14}$	0	$\frac{-f_{XI}\cdot i_{N,XI}+i_{N,BM}}{14}$	0	f_{XI}	-1	0	0	0	0
10	p7(b)	Anoxic Endog. Resp. of X_H NO_2-N_2	0	0	$-f_{XI}\cdot i_{N,XI}+i_{N,BM}$	$-\frac{1-f_{XI}}{1.72}$	0	$\frac{1-f_{XI}}{1.72}$	$\frac{i_{N.BM}-f_{XI}\cdot i_{N,XI}-(f_{XI}-1)/1.72}{14}$	0	f_{XI}	-1	0	0	0	0
11	p8	Aerobic Endog. Resp. of X_{STO}	-1	0	0	0	0	0	0	0	0	0	0	-1	0	0
12	p9(a)	Anoxic Endog. Resp. of X_{STO} NO_3-NO_2	0	0	0	$\frac{1}{1.14}$	$-\frac{1}{1.14}$	0	0	0	0	0	0	-1	0	0
13	p9(b)	Anoxic Endog. Resp. of X_{STO} NO_2-N_2	0	0	0	$-\frac{1}{1.72}$	0	$\frac{1}{1.72}$	$\frac{1}{24.08}$	0	0	0	0	-1	0	0
14	p10(a)	Aerobic Growth of X_{AOB}, Nitritation	$1-\frac{48}{14\cdot Y_{AOB}}$	0	$-\frac{1}{Y_{AOB}}-i_{N,BM}$	$\frac{1}{Y_{AOB}}$	0	0	$-\frac{1}{14}(\frac{2}{Y_{AOB}}+i_{N.BM})$	0	0	0	0	0	1	0
15	p11(a)	Aerobic End. Resp. of X_{AOB}	-(1- f_{XI})	0	$-f_{XI}\cdot i_{N,XI}+i_{N,BM}$	0	0	0	$\frac{-f_{XI}\cdot i_{N,XI}+i_{N,BM}}{14}$	0	f_{XI}	0	0	0	-1	0
16	p12(a)	Anoxic End. Resp. of X_{AOB}	0	0	$-f_{XI}\cdot i_{N,XI}+i_{N,BM}$	0	$-\frac{1-f_{XI}}{2.86}$	$\frac{1-f_{XI}}{2.86}$	$\frac{i_{N.BM}-f_{XI}\cdot i_{N,XI}-(f_{XI}-1)/2.86}{14}$	0	f_{XI}	0	0	0	-1	0
17	p10(b)	Aerobic Growth of X_{NOB}, Nitratation	$1-\frac{16}{14\cdot Y_{AOB}}$	0	$-i_{N,BM}$	$-\frac{1}{Y_{NOB}}$	$\frac{1}{Y_{NOB}}$	0	$-\frac{i_{N,BM}}{14}$	0	0	0	0	0	0	1
18	p11(b)	Aerobic End. Resp. of X_{NOB}	-(1- f_{XI})	0	$-f_{XI}\cdot i_{N,XI}+i_{N,BM}$	0	0	0	$\frac{-f_{XI}\cdot i_{N,XI}+i_{N,BM}}{14}$	0	f_{XI}	0	0	0	0	-1
19	p12(b)	Anoxic End. Resp. of X_{NOB}	0	0	$-f_{XI}\cdot i_{N,XI}+i_{N,BM}$	0	$-\frac{1-f_{XI}}{2.86}$	$\frac{1-f_{XI}}{2.86}$	$\frac{i_{N.BM}-f_{XI}\cdot i_{N,XI}-(f_{XI}-1)/2.86}{14}$	0	f_{XI}	0	0	0	0	-1

Table S4. Kinetic and stoichiometric parameters at 20 ℃.

Index	Symbol	Characterization	Low	Value	High	θ	Units	references
	$i_{N,SI}$	N content of inert soluble COD S_I	0.005	0.01	0.01		[g N/g COD]	[1]
1	$i_{N,SS}$	N content of readily biodegradable substrate	0.01	0.03	0.05		[g N/g COD]	[1]
2	$i_{N,XI}$	N content of inert particulate COD X_I	0.01	0.04	0.07		[g N/g COD]	[1]
	$i_{N,XS}$	N content of slowly biodegradable substrate	0.01	0.03	0.05		[g N/g COD]	[1]
3	$i_{N,BM}$	N content of biomass X_H, X_{AOB}, X_{NOB}	0.03	0.07	0.11		[g N/g COD]	[1]
	f_{SI}	Production of S_I in hydrolysis	0.00	0.00	0.02		[g COD/g COD]	[2]
4	f_{XI}	Fraction of inert COD generated in biomass lysis	0.1	0.2	0.3		[g COD/g COD]	[1]
5	Y_{H,O_2}	Yield coeff. for heterotrophs in aerobic growth	0.65	0.8	0.95		[g COD/g COD]	[1]
6	Y_{H,NO_3}	Yield coeff. for heterotrophs in anoxic growth (NO_3^--N- NO_2^--N)	0.5	0.65	0.8		[g COD/g COD]	[1]
7	Y_{H,NO_2}	Yield coeff. for heterotrophs in anoxic growth (NO_2^--N- N_2-N)	0.5	0.65	0.8		[g COD/g COD]	[1]
8	Y_{STO,O_2}	Yield coeff. for X_{STO} in aerobic growth	0.2	0.8	1		[g COD/g COD]	[1]
9	Y_{STO,NO_3}	Yield coeff. for X_{STO} in anoxic growth (NO_3^--N- NO_2^--N)	0.2	0.7	1		[g COD/g COD]	[1]
10	Y_{STO,NO_2}	Yield coeff. for X_{STO} in anoxic growth (NO_2^--N- N_2-N)	0.2	0.7	1		[g COD/g COD]	[1]
11	Y_{AOB}	Yield coeff. for X_{AOB}	0.1	0.18	0.3		[g COD/g N]	[3]
12	Y_{NOB}	Yield coeff. for X_{NOB}	0.02	0.06	0.1		[g COD/g N]	[3]
13	k_H	Hydrolysis rate constant	4	9	15	0.04	[d^{-1}]	[1]
14	k_{STO}	Maximum storage rate	4	12	20	0.07	[d^{-1}]	[1]
15	μ_H	Maximum growth rate on substrate of heterotrophs	1	3	6	0.07	[d^{-1}]	[1]
16	μ_{AOB}	Maximum growth rate of X_{AOB}	0.8	0.9	1.1	0.12	[d^{-1}]	[3]
17	μ_{NOB}	Maximum growth rate of X_{NOB}	0.5	0.65	1.0	0.078	[d^{-1}]	[3]
18	b_{H,O_2}	Aerobic end. resp. rate for X_H	0.1	0.3	0.5	0.07	[d^{-1}]	[1]
19	b_{STO,O_2}	Aerobic end. resp. rate for X_{STO}	0.1	0.3	0.5		[d^{-1}]	[1]
20	b_{AOB}	Decay rate of X_{AOB}	0.05	0.15	0.25	0.12	[d^{-1}]	[4]
21	b_{NOB}	Decay rate of X_{NOB}	0.1	0.22	0.34	0.078	[d^{-1}]	[4]
22	η_{H,NO_3}	Reduction factor for denitrification (NO_3^--N- NO_2^--N)	0.1		1		-	[3]
23	η_{H,NO_2}	Reduction factor for denitrification (NO_2^--N- N_2-N)	0.1		1		-	[3]
24	$\eta_{H,end.NO_3}$	Reduction factor for b_H, anoxic condition (NO_3^--N- NO_2^--N)	0.25		1		-	[3]
25	$\eta_{H,end.NO_2}$	Reduction factor for b_H, anoxic condition (NO_2^--N- N_2-N)	0.35		1		-	[3]
26	$\eta_{N,end}$	Reduction factor for b_{AOB} and b_{NOB}, anoxic condition	0.1		1		-	[4]
27	K_X	Hydrolysis saturation constant	0.2	1.0	2		[g X_S g^{-1} X_H]	[1]
28	K_{H,O_2}	Saturation coeff. for oxygen, het. growth	0.05	0.2	0.5		[g O_2 m^{-3}]	[1]
29	$K_{H,O_2.inh}$	Inhibition coeff. for oxygen, het. growth	0.05	0.2	5		[g O_2 m^{-3}]	[1]
30	$K_{H,SS}$	Saturation coeff. for readily biodegradable substrates, het. growth	5	10	20		[g COD m^{-3}]	[1]
31	K_{H,NH_4}	Saturation/inhibition coefficient for ammonium, het. growth	0.001	0.01	0.1		[g N m^{-3}]	[1]
32	K_{H,NO_3}	Saturation/inhibition coefficient for nitrate, het. growth	0.1	0.5	10		[g N m^{-3}]	[1]
33	K_{H,NO_2}	Saturation/inhibition coefficient for nitrite, het. growth	0.1	0.5	10		[g N m^{-3}]	[1]
34	$K_{H,ALK}$	Saturation/inhibition coefficient for alkalinity, het. growth	0.01	0.1	1		[mole HCO_3^- m^{-3}]	[1]
35	$K_{H,STO}$	Saturation coeff. for storage products	0.01	0.1	1		[g COD m^{-3}]	[1]
36	K_{AOB,O_2}	Saturation coefficient for oxygen, AOB	0.2	0.8	5		[g O_2 m^{-3}]	[5]
37	K_{NOB,O_2}	Saturation coefficient for oxygen, NOB	0.2	0.8	5		[g O_2 m^{-3}]	[5]
38	K_{AOB,NH_4}	Saturation coeff. for ammonium, AOB	0.05	0.14	0.5		[g N m^{-3}]	[5]
39	K_{NOB,NO_2}	Saturation coefficient for nitrite, NOB	0.05	0.28	0.5		[g N m^{-3}]	[5]
40	$K_{N,ALK}$	Saturation/inhibition coefficient for alkalinity, aut. growth	0.1	0.5	1		[mole HCO_3^- m^{-3}]	[1]

References:

1. Koch G, Kuhni M, Gujer W, Siegrist H. Calibration and Validation of Activated Sludge Model no. 3 for Swiss Municipal Wastewater. *Water Research* **34**, 3580-3590 (2000).

2. Gujer W, Henze M, Mino T, van Loosdrecht M. Activated sludge model No. 3. *Water Science and Technology* **39**, 183-193 (1999).

3. Kaelin D, Manser R, Rieger L, Eugster J, Rottermann K, Siegrist H. Extension of ASM3 for two-step nitrification and denitrification and its calibration and validation with batch tests and pilot scale data. *Water Research* **43**, 1680-1692 (2009).

4. Manser R, Gujer W, Siegrist H. Decay processes of nitrifying bacteria in biological wastewater treatment systems. *Water Research* **40**, 2416-2426 (2006).

5. Manser R, Gujer W, Siegrist H. Consequences of mass transfer effects on the kinetics of nitrifiers. *Water Research* **39**, 4633-4642 (2005).
